# Data on Swiss public's acceptance and sustainability perceptions of food produced with chemical, digital and mechanical weed control measures and the influence of information source on technology perception in agriculture

**DOI:** 10.1016/j.dib.2024.111212

**Published:** 2024-12-10

**Authors:** Jeanine Ammann, Nadja El Benni, Sandie Masson, Rita Saleh

**Affiliations:** a Agroscope, Economic Modelling and Policy Analysis Group, Tänikon 1, 8356 Ettenhausen, Switzerland; b Agroscope, Research Division Sustainability Assessment and Agricultural Management, Tänikon 1, 8356 Ettenhausen, Switzerland; c Agroscope, Weed Science for Field Crops Group, Route de Duillier 50, 1260 Nyon, Switzerland; d Agroscope, Socio-Economics Group, Tänikon 1, 8356 Ettenhausen, Switzerland

**Keywords:** Public acceptance, Sustainability perceptions, Weed control, Herbicide use, Chemophobia, Perception of farmers

## Abstract

This article describes data from an online survey conducted with the Swiss public from the two biggest language regions (German and French) in Switzerland. The survey was conducted in February 2023. Participants were recruited through a professional panel provider and quotas were used for age, gender and language region. The final sample contained 485 respondents. In the first part of the survey, respondents provided basic sociodemographic information. In the second part, their sustainability perceptions regarding four different weed management practices (full-surface spraying, hoeing machine, spot spraying and precise spraying) were investigated. Respondents were then randomly assigned to one of five experiment groups, in which information on a hoeing and a milking robot was presented, using 5 different information sources (male/female farmer, male/female scientist, no source). Technology perception was assessed using several questions and aspects (e.g. perception of economic, environmental and social sustainability). Finally, respondents answered questions assessing their attitudes towards the perception of farmers, food technology neophobia, chemophobia and the importance of naturalness. The survey can be used and adapted to different contents, aiming to investigate public perception of smart farming technologies and the influence of information sources on technology perception.

Specifications Table

**Table:** 

Subject	*Social Sciences*
Specific subject area	*Public sustainability perception of farming technologies and acceptance of food produced with chemical, digital and mechanical weed control measures and the influence of information source*
Type of data	*Cleaned, raw*
Data collection	*Participants were recruited by Bilendi AG (ISO-certified panel provider) and the data were collected through an online survey (accessible from computer and phone) implemented with Tivian. Data collection took place in the German- and French-speaking parts of Switzerland in February 2023. Quotas were used for age, gender and language region.*
Data source location	*Institution: Agroscope* *City/Town/Region: Ettenhausen, Tänikon* *Country: Switzerland*
Data accessibility	Repository name: Zenodo Data identification number: *10.5281/zenodo.10817295* Direct URL to data: https://zenodo.org/records/10817295 [1]
Related research article	Saleh, R., El Benni, N., Masson, S., & Ammann, J. (2024). Public acceptance and sustainability perceptions of food produced with chemical, digital and mechanical weed control measures. *Food Quality and Preference, 113*. [2] https://www.sciencedirect.com/science/article/pii/S0950329323002732?via%3Dihub

## 1 Value of the Data

• The data describe how the Swiss public in the two biggest language regions perceives sustainability of food produced using chemical, digital and mechanical weed control measures. With that, the data can help a better understanding of technology perception and acceptance.
• The data show that communicating information on the quantity of herbicide applied and the precision of the spraying can ensure public acceptance of the chemical-based weed control measures, which ultimately secures public support to farmers’ adoption of the digital technologies (i.e., spot-spraying, precision spraying).
• The data investigate possible influences of information source in farm technology communication. With that, the data can help inform technology communication.
• The survey can be used for use in other contexts or countries and thereby enable cross-cultural comparisons.

## 2 Background

Using digital technologies can help tackle sustainability challenges in agriculture [3,4].High technology costs can be a significant barrier for farmers to adopt new technologies [5]. At the same time, technological development leads to a decrease in technology costs. For crop farming, technology use can lower the use of pesticides and therefore the related costs [3]. At the same time, public interest in sustainability has increased, associating it predominantly with environment-friendly food production requiring few to no chemical use [6]. However, the public is concerned with chemical use in food production (i.e., the use of pesticides and herbicides) as they consider it not only harmful to the environment but also to their health [7]. These concerns are depicted in literature influencing public acceptance of chemical use in food technologies [6,[8], [9], [10]].Furthermore, people may reject conventional use of chemicals, as well as any farming technologies which relies on chemical use [7,11,12]. They might even support political initiatives banning pesticide and herbicide use and favoring farming technologies and practices that do not require chemical use such as mechanical interventions [13].

With this dataset, we aimed to identify the changes and inter-relationships in people's perceptions of the social, economic and environmental sustainability of farming technologies that reduce reliance on or eliminates entirely the use of chemicals in food production. For this, we focus on the weed control measures as they are the most well-established in terms of reducing and eliminating chemical use [14,15]. More specifically, we examined four weed control measures. Full-surface spraying uses a traditional sprayer, that applies herbicides to the entire field surface. We also examined two measures that measures which entail site-specific spraying of herbicides ensures a substantial reduction in the amounts of herbicides use compared to conventional full-surface herbicide spraying [16,17]. Spot spraying and precise spraying rely on artificial intelligence systems that detect weeds and localise spraying [18,19]. In precise spraying, only weeds are sprayed, while in spot spraying, the zones of crops containing weeds are targeted. Herbicide-free technologies, such as the hoeing robot, rely entirely on mechanical functions to remove weeds. For example, hoeing machines are tractors pulling a series of horizontal hoes that aerate the soil while simultaneously uprooting weeds in the inter-rows [20]. Moreover, our data includes information regarding changes in acceptance and naturalness perceptions of the described technologies. This allows to investigate how individual factors (sustainability perceptions, trust in farmers, chemophobia) influence the acceptability of these measures.

## 3 Data Description

Data were collected through an online survey in Switzerland in February 2023. Participant recruitment was done by a professional panel provider (Bilendi AG). We used quotas for gender (50 % women), age (33 % aged 18–35, 33 % aged 36–54, and 33 % aged 55–75), and language region (50 % German, 50 % French). For each of the two language regions, we aimed to recruit 250 participants.

In total 542 respondents completed the survey. We excluded 57, as their participation duration was below half of the median for the total sample. The final sample consisted of 485 respondents (51.1 % women, mean age = 46 years, standard deviation = 15 years, range = 20–75 years). The respondents’ educations ranged from basic (4.7 %) to intermediate (44.3 %) to advanced (50.9 %). Finally, 43.3 % of participants indicated that they resided in rural areas, 22.3 % in suburban areas and 34.4 % in urban areas.

Due to the survey design, participants were required to enter a response in order to proceed with the survey. As a result, there were no missing variables in the dataset. The dataset in wide format after data cleaning (cleaned; CSV), the survey in three languages (German, French and English; PDF) and the codebook describing the variables (PDF) are freely available online through Zenodo: https://zenodo.org/records/10817295 [1].

## 4 Experimental Design, Materials and Methods

Our survey was programmed and conducted using the online survey tool, Unipark (Management Questback GmbH, Germany). It consisted of the following parts:

1. Language selection (German / French)
2. Introduction and consent
3. Sociodemographic questions
4. Technology perception
5. Information sources
6. Perception of farmers
7. Food technology neophobia
8. Chemophobia
9. Importance of naturalness
10. Thank you and end

In the first part of the survey, participants could choose their preferred language (German or French). Next, participants were briefly informed about the contents of the survey and provided their informed consent, following internal guidelines [21]. We further informed them that they were free to quit the survey at any time without having to give a reason.

In the third part of the survey, sociodemographic information was obtained (see Table 1). This included participants’ age, gender, education level (covering the education system in Switzerland), place of living (rural vs. urban, in accordance with terms used by the Swiss Statistical Office [22]) and political orientation. For political orientation, we used an interactive slider scale from “very left” (0) to “very right” (100), which was done similarly other studies including Eurobarometer [23].

**Table 1:** Sociodemographic information of the sample (N = 485).

		Language	
		German	French
		n = 244 M (SD) or [%]	n = 241 M (SD) or [%]
Age		46.2 (15.1)	46.3 (14.9)
Political orientation		50.6 (20.8)	55.0 (22.0)
Gender			
	Women	51.2	51.0
	Men	48.8	49.0
Education level			
	Mandatory school (primary, secondary school)	3.3	4.1
	Basic apprenticeship, voluntary social year, prevocational school	0	2.1
	Apprenticeship	46.7	28.6
	High school	5.3	7.9
	Technical and vocational training	20.1	11.6
	University of applied science	10.7	29.9
	University or ETH	13.9	15.8
Place of living			
	Rural	7.8	11.6
	Village	40.2	27.0
	Small town	25.8	18.7
	Medium-sized city	13.9	20.7
	Large city	12.3	22.0

The fourth part of the survey was intended to assess participants’ technology perception. For this, participants read the description of the weed control problem, its negative consequences on crops and the need for management. Subsequently, they read descriptions of the four weed control measures (i.e. full surface spraying, spot spraying, precise spraying and hoeing machine). The order of presentation for these four measures was randomized to control for possible order effects. All descriptions were developed by agricultural experts and tested by a non-experts to make sure they were clear and understandable. To ensure that the measures were evident to the respondents, both a written description and an image were presented. The images focused on the technologies, showing the tractors and sprayers or hoeing machine. They did not depict specific crops to control for potential crop-specific effects. The descriptions used read as follows:

***Full-surface spraying****: The application of herbicides with the traditional sprayer is a chemical weed control measure. Herbicides are applied to the entire field surface, without distinguishing between weeds and crops. Herbicides only act on weeds.*
***Hoeing machine:****The hoeing machine is a mechanical weeding measure. The hoeing machine's crowfoot shares pull weeds between the crop rows by penetrating the soil at the roots. At the same time, the softer rotating fingers prune the weeds in the row without damaging the crops.*
***Spot spraying:****The targeted application of herbicide by section cutting on the sprayer is a digitally controlled weed control measure. Cameras in the tractor detect weeds, and the information is transmitted to the sprayer using artificial intelligence. In areas where weeds are identified, only one section of nozzles delivers a precise amount of herbicides. The crop plants also receive the herbicides, but the latter does not have an effect on them.*
***Precise spraying:****The application of herbicides with a precision sprayer is a digitally controlled measure of weed control. The machines used for application distinguish between crop plants and unwanted weeds using cameras and artificial intelligence directly in the sprayer. A precise amount of herbicides is accurately sprayed by individually controlled nozzles only on the identified weeds. Crop plants do not receive the herbicides.*

For each of the four technologies, participants answered six questions using an interactive slider scale from 0 to 100 to provide their answers. The questions were presented in random order. In one question, respondents indicated their acceptance of food produced using each measure by specifying their willingness to consume food produced using these measures on a scale from 0 (not willing at all) to 100 (completely willing). Further, they were asked how effective the measure was in their view in controlling the weed and how natural the food grown using this technology was. Further, respondents indicated how sustainable they perceived the measures to be on social, environmental and economic dimensions each, using a scale from 0 (not sustainable at all) to 100 (completely sustainable). Results are presented in Table 2. For this measurement, the respondents were provided with short definitions of the sustainability dimensions to ensure a common understanding. The definitions used read as follows:

***Environmental sustainability****in agriculture refers to the good stewardship of natural resources to avoid or reduce negative impacts on the environment.*
***Economic sustainability****in agriculture refers to managing a farm in a way that ensures long-term profitability.*
***Social sustainability****in agriculture refers to good farm work conditions in terms of health and acceptable labour in a way that ensures the farmers’ long-term work and livelihood satisfaction.*

**Table 2:** Mean (M), standard deviation (SD) and quartiles (25, 50 and 75) for various aspects regarding the four weed control measures investigated (N = 485).

		Weed control measures M (SD) [quartiles]			
		Full-surface spraying	Spot spraying	Precise spraying	Hoeing machine
	Acceptance of food produced using the respective measure	43.31 (29.56) [18, 44, 67]	50.93 (27.69) [28.5, 52, 70]	58.01 (29.29) [39, 62, 82]	83.99 (18.92) [74, 91, 99]
	Effectiveness for weed control	64.03 (26.74) [49, 70, 85]	63.36 (24.47) [50, 67, 81]	66.39 (24.77) [51, 70, 85.5]	70.27 (20.38) [55, 71, 86]
	Naturalness perception	34.69 (28.13) [8.5, 31, 55]	44.06 (26.52) [23, 47, 63]	50.81 (28.67) [27.5, 52, 74]	81.95 (20.13) [72, 89, 99]
Sustainability perception					
	Environmental	36.82 (28.85) [10, 34, 58]	46.88 (26.79) [26.5, 49, 67.5]	52.95 (29.04) [31, 56, 76]	79.92 (19.27) [69, 84, 97]
	Social	42.31 (27.66) [19, 44, 63.5]	50.94 (25.93) [32, 50, 70]	56.07 (27.61) [36.5, 58, 78]	76.34 (20.72) [62.5, 80, 95]
	Economic	51.49 (28.70) [29, 51, 75]	54.85 (24.74) [41, 55, 73]	58.77 (26.48) [43, 61, 79]	72.92 (20.50) [58, 75, 91]

Note. All aspects were rated on a scale from 0 (not at all) to 100 (very much).

For the next part of the survey, participants were randomly assigned to one of five experiment groups, where they read a short description of a technology (hoeing robot and milking robot) and the person using it (female/male, farmer/scientist, no information; see Table 3). Each group read two scenarios (i.e. the hoeing robot and the milking robot scenario). For each scenario, participants answered five questions. These were answered on an interactive slider scale from 0 to 100. For the hoeing robot (scenario 1), respondents were asked how acceptable they perceived the use of the described technology to be in terms of workload, environmental-friendliness and cost/ benefit. Further, they were asked how willing they were to consume vegetables produced with the use of this technology, how trustworthy they thought the technology was and what type of feelings the technology evoked.

**Table 3:** Overview on the five experiment groups used in the part of the survey where the influence of information source on technology perception was investigated.

	Group 1	Group 2	Group 3	Group 4	Group 5
	Female farmer	Male farmer	Female scientist	Male scientist	No person (control)
**Scenario 1: Hoeing robot**					

	Maria / Manuel is a farmer, who uses the hoeing robot to control weeds to reduce labour and increase efficiency. Hoeing robots are autonomous mechanical robots that control weeds by mechanical hoeing. One advantage of weed control with hoeing robots is that the health of the crop can be monitored and ensured at the same time.		Maria / Manuel is a scientist, who uses the hoeing robot to control weeds to reduce labour and increase efficiency. Hoeing robots are autonomous mechanical robots that control weeds by mechanical hoeing. One advantage of weed control with hoeing robots is that the health of the crop can be monitored and ensured at the same time.		A weeding robot is used on a vegetable farm to reduce labour and increase efficiency. Hoeing robots are autonomous mechanical robots that control weeds by mechanical hoeing. One advantage of weed control with hoeing robots is that the health of the crop can be monitored and ensured at the same time.

**Scenario 2: Milking robot**					

	Manuela / Manuel is a farmer, who uses milking robots on her / his dairy cows to reduce workload and increase efficiency. Milking robots are autonomous systems for milking cows. One advantage of this milking system is that the cows can decide for themselves when they want to be milked and their health is monitored at the same time.		Manuela / Manuel is a scientist, who uses milking robots on her / his dairy cows to reduce workload and increase efficiency. Milking robots are autonomous systems for milking cows. One advantage of this milking system is that the cows can decide for themselves when they want to be milked and their health is monitored at the same time.		Milking robots are used on dairy cows to reduce workload and increase efficiency. Milking robots are autonomous systems for milking cows. One advantage of this milking system is that the cows can decide for themselves when they want to be milked, and their health is monitored at the same time.

For the milking robot (scenario 2), respondents were asked how acceptable they perceived the use of the described technology to be in terms animal health and welfare, workload, environmental-friendliness and cost/ benefit. Further, to get a better understanding on participants’ technology perception, they were asked how willing they were to consume milk produced with the use of this technology, how trustworthy they thought the technology was and what type of feelings the technology evoked. Trust in the technology was included as it can be an important dimension of consumers’ sustainability perception [24].

For perceptions of farmers, participants indicated how strongly they agreed with each of five items on a seven-point Likert scale from 1 (do not agree at all) to 7 (totally agree) [25]. The items cover aspects such as animal welfare, environmental consciousness and general attitudes towards farmers. The item-total correlations (>0.5) for the items of this scale indicated that the items were consistent within each other in measuring the constructs of interest. Cronbach's alpha (.84) indicated that the scale was reliable.

Food technology neophobia was measured using 13 items according to Cox and Evans [26]. These items were rated for agreement on a scale from 1 (do not agree at all) to 7 (totally agree).

For chemophobia, we used the scale developed by Saleh et al. (2021) [8], where participants indicated how strongly they agreed with each of five items using a six-point Likert scale from 1 (strongly disagree) to 6 (strongly agree). The items cover perception of chemicals and perceived risks related to chemicals.

For the importance of food naturalness, participants indicated how important it was to eat food with no additives or artificial ingredients and containing only natural ingredients on a scale ranging from 1 (not important at all) to 4 (very important) following Steptoe et al. (1995) [27].

In the final part of the survey, participants had the possibility to write down any comments if they wished to do so. After that, we thanked participants for their participation and they were instructed to close the survey.

## Limitations

An important limitation of our study is that social sustainability was defined in terms of farmers’ health and working conditions. The definition did not incorporate other important social aspects, such as farmer-consumer interactions [28]. Another limitation is the fact that we presented the weed control measures individually to respondents, which hinders any conclusions regarding their acceptance of the widely used integrated weed management measures which relies on a combination of the investigated measures.

## Ethics Statement

The researchers adhered to all ethical considerations during the data collection process and followed institutional [21] and psychological ethical guidelines [29]. All participants involved in the study provided their written, informed consent to participate. Participation was voluntary and could be withdrawn at any time. Participants remained anonymous and their responses were dealt with in confidence.

## Credit Author Statement

**Jeanine Ammann:** Conceptualization, Methodology, Software, Data curation, Formal analysis, Writing – original draft. **Nadja El Benni:** Conceptualization, Writing – review & editing. **Sandie Masson:** Conceptualization, Writing – review & editing. **Rita Saleh:** Conceptualization, Methodology, Software, Data curation, Formal analysis, Visualization, Writing – review & editing.

## Declaration of Competing Interest

The authors declare that they have no known competing financial interests or personal relationships that could have appeared to influence the work reported in this paper.

## Data Availability

ZenodoSwiss public's acceptance and sustainability perceptions of food produced with chemical, digital and mechanical weed control measures and the influence of information source on technology perception i (Original data).
